# Restless Leg Syndrome in Diabetics Compared with Normal Controls

**DOI:** 10.1155/2014/871751

**Published:** 2014-05-07

**Authors:** Mehdi Zobeiri, Azita Shokoohi

**Affiliations:** ^1^Department of Internal Medicine, School of Medicine, Imam Reza Hospital, Kermanshah University of Medical Sciences, Kermanshah, Iran; ^2^Kermanshah University of Medical Sciences, Kermanshah, Iran

## Abstract

*Introduction*. Restless leg syndrome (RLS) is a common sleep disorder which is characterized by urge to move the legs accompanied by disturbing and uncomfortable leg sensation during night and rest. This common condition affects 7–10% of general population and is frequently unrecognized, misdiagnosed, and poorly managed. Several clinical conditions like diabetes have been associated with secondary form of RLS. This study analyzed the frequency and possible risk factor for RLS development in diabetic patient. *Material and Methods*. This descriptive case-control study was done on 140 consecutive outpatient diabetics and age, sex, and body mass index matched control group. RLS was diagnosed by criteria of the International RLS Study Group. *Results*. Prevalence of RLS was 28.6% in diabetes and 7.1% in control group (*P* = 0.001). Sex difference was not significant and with rising duration of diabetes prevalence of RLS was not increased. *Discussion*. With regarding significant association between RLS and diabetes and its negative impact on quality of life/health outcome/sleep/daytime activity/cognitive function/ and mental state of diabetic patient/higher awareness of RLS among physicians and related health worker suggested.

## 1. Introduction

Restless legs syndrome (RLS) is a common sleep disorder characterized by unpleasant night sensations (tingling, creeping) in the legs and rarely arms that are temporarily relieved by movement and leads to a severe difficulty in initiating and maintaining sleep [1–3].

RLS typically worse during periods of rest, relaxation, or inactivity and may be accompanied with major impact on daytime function and quality of life [2, 3]. RLS is a common disease that occurs in 7–10% of general population, increasing with age and affecting women more often than men and parity is a major factor in explaining the sex difference [1, 4–6].

RLS is often familial or idiopathic, recognized as primary but may be associated with, renal failure, iron deficiency, rheumatoid arthritis, polyneuropathy, cryoglobulinemia, and infection [6]. Primary RLS is familial in up to two-thirds of patients believed to be an autosomal dominant disorder and secondary form is most common in those presenting for the first time in later life [7, 8].

This neurological problem despite trials for better recognition remains an undiagnosed clinical condition [4, 9]. Diagnosis of RLS is purely clinical and there is no specific test [2, 7].

The essential clinical diagnostic criteria for restless legs syndrome were developed and approved by workshop participants and the executive committee of the International Restless Legs Syndrome Study Group as specified by the National Institutes of Health, and all four essential criteria must be met for a positive diagnosis in more than five times per month [2, 10]. Diabetic patients have 4–4.4-time more risk of developing RLS than in the general population although in one study no relation detected [2, 6, 11]. Significant association between RLS and diabetes is not wonderful because of etiologic role of diabetes in producing polyneuropathy and renal failure [10]. The aims of this investigation were to look for an association between RLS and diabetes in a case-control study and to identify possible risk factors for the development of RLS in diabetic patients.

## 2. Material and Methods

This is a case-control study between 140 consecutive patients with diabetes attending the diabetes center of the Kermanshah University Hospital, which were recruited from March 2007 to July 2007 and the control group consists of 140 patients without diabetes who were admitted in the ENT department. Data collection was done by physician from check list which includes demographic information, variables related to diabetes, and diagnostic criteria of RLS which were assessed with standardized, validated questions addressing the 4 minimal criteria for RLS as defined by International Restless legs Syndrome Study Group. Both groups were matched based on age, sex, and body mass index (BMI). Exclusion criteria were renal disease, iron deficiency anemia, rheumatoid arthritis, and pregnancy. In diabetic patient, disease duration and type of diabetes determined but polyneuropathy were not assessed. Data were analyzed by use of two dimensional frequency tables and calculation of tchouprov qualitative correlation coefficient for relation of risk factors and diabetes type. For summarizing quantitative variables, data are displayed in tables as means and standard deviations and *Z* test was used for comparison of RLS prevalence between two groups. In order to matching between two groups independent chi-square and as needed Fisher's exact test and Mann-Whitney *U* test were used.

## 3. Results

Mean age in diabetic was 46.3 ± 13.93 and in control group was 44.02 ± 19.1 (*P* = 0.252). Mean BMI in diabetic was 25.19 ± 3.73 and in control group was 24.61 ± 3.11 (*P* = 0.159). In each group, 80 (57.1%) were female and 60 (42.9%) were male. As a whole RLS prevalence was 17.9% which is 28.6% in diabetic and 7.1% in control group (*P* = 0.001).

General characteristics of RLS frequency in the diabetic patients and of the nondiabetic controls are reported in Table 1 (more than 5 times per month regarded as positive RLS symptom).

Between related variables in diabetic and control, only hypertension was significantly higher in diabetic groups (Table 2).

Characteristics of variables in both diabetic and control groups with and without RLS are depicted in Table 3.

The only difference was hypertension, which was significantly higher among RLS groups.

## 4. Discussion

The prevalence of RLS in our diabetic patients (28.6%) was significantly higher than nondiabetic controls (7.1%), whereas it was higher with respect to previous studies carried out in subjects with type 2 diabetes [10]. The studies are consistent with the idea that RLS is a common condition, at least in populations derived from Western Europe and USA with prevalence between 5.8 and 11.4% [12]. A number of epidemiological studies of RLS prevalence from Asia found lower prevalence in Japanese (2–4%) and Singapore (0.1%) populations [13, 14].

IRAN and its western parts like Kermanshah city have peoples belonging to Caucasian race [15].

More than four times higher prevalence of RLS in diabetic than nondiabetic control suggests strong association between RLS and diabetes. Because of some association between neuropathy and RLS, increased risk for RLS in diabetic may reflect partial consequences of diabetic neuropathy rather than diabetes per se [16].

Cho et al. show more than two time higher confirmed RLS prevalence in diabetics than control with osteoarthritis through face-to-face interviews using the 18-item Hopkins Diagnostic Questionnaire, which removes RLS mimics [16].

Polyneuropathy is a risk factor for RLS in diabetic patients but after adjusting for the presence of polyneuropathy diabetes remains an independent risk factor for RLS [8].

In this study, polyneuropathy was not evaluated and RLS symptoms were assessed as a whole. Studies have documented the relative iron stores depletion and impaired dopaminergic neurotransmission of brain which is a probable pain control system in RLS patients [12, 17]. Iron is needed for dopamine synthesis and, at least in animal models, iron deficiency during early life can result in lifetime abnormalities of the dopamine system [6, 12].

In the diabetic population, RLS seems to be independent from the iron status of patients [8, 10]. Hypertension was higher in diabetic and control group with RLS which seems to be partially related to RLS.

Mean age of study groups suggest secondary form of RLS which is most common in those presenting for the first time in later life [6]. Women were not significantly affected than men although in two studies significant higher prevalence of women was found and parity defined as a major factor in explaining the sex difference [1, 6]. Prevalence of RLS was not increased with rising duration of diabetes, while there was witnessed increases with age [6].

RLS as a sleep disorder may have impact on diabetes management and health outcome [17, 18].

It is associated with impairs sleep quality, drug consumption at night, daytime activity, cognitive function, and depressive and anxious symptoms and may be a risk factor for hypertension and cardiovascular disease [17, 19, 20].

RLS as a one of the most intriguing chronic sensory-motor disorders is frequently unrecognized, misdiagnosed, and poorly managed [8, 21]. Awareness about RLS is poor among medical professionals and diagnosis of RLS was missed not only by general physicians, but also by specialists like neurologists and psychiatrists [22–24]. RLS symptoms are not reported by patients to their health care providers. So, identification and suitable management of RLS, especially in diabetic populations, are recommended [8, 21].

## Figures and Tables

**Table 1:** RLS frequency in diabetic patients and of the nondiabetic controls.

RLS symptom	Study groups		
	Diabetic	Control	Total
Never	52 (37.1%)	84 (60%)	136 (48.6%)
Seldom (one time per month)	19 (13.6%)	23 (16.4%)	42 (15%)
Sometimes (2–4 times per month)	29 (20.7%)	23 (16.4%)	52 (18.6%)
Often (5-6 times per month)	25 (17.9%)	7 (5%)	32 (11.4%)
Always (≥16 times per month)	15 (10.7%)	3 (2.1%)	18 (6.4%)

Total	140 (100%)	140 (100%)	280 (100%)

**Table 2:** Frequency of related variables in diabetic patients and of the nondiabetic controls.

Variables	Groups		
	Diabetic	Control	*P*
Smoking	22	9	0.013
Alcohol consumption	2	1	0.562
Hypertension	40	18	0.001
Exercise	82	28	0.001
(24.9) BMI mean	3.73 ± 25.19	3.11 ± 24.61	0.159
Mean age	13.93 ± 46.31	19.10 ± 44.02	0.252

**Table 3:** Comparison of related variable in groups with and without RLS.

variables	Groups		
	Without RLS (%)	With RLS (%)	*P*
Smoking	23 (10)	8 (16)	0.22
Alcohol consumption	2 (0.86)	1 (2)	0.428
Hypertension	40 (1.73)	18 (36)	0.003
Exercise	86 (37.3)	24 (48)	0.164
(24.9) BMI mean	(24.6 ± 3.3)	(25.9 ± 3.6)	0.18

Total	230	50	

RLS was not significantly more common in female than male (20% versus 15%) (*P* = 0.280). 27% of diabetic patients with ≤10 years and 34% with ≥10 years diabetic duration had RLS (*P* = 0.429).
